# Assessment of human gait after total knee arthroplasty by dynamic time warping algorithm

**DOI:** 10.1049/htl2.12047

**Published:** 2023-06-13

**Authors:** Reza Abbasi‐Kesbi, Mohammad Fathi, Mohammad Najafi, Alireza Nikfarjam

**Affiliations:** ^1^ MEMS & NEMS Department, Faculty of New Sciences and Technologies University of Tehran Tehran Iran; ^2^ Department of Biomedical Engineering, Faculty of Medical Sciences and Technologies Islamic Azad University Science and Research Branch Tehran Iran

**Keywords:** biomedical measurement, gait analysis, motion measurement

## Abstract

Today, the elderly population is increasing, and there are many drawbacks for them, especially defects in their knee joints which lead to improper gait. To solve this problem, their knee joint can be replaced with knee arthroplasty. In this letter, level of improvement in the human gait before and after total knee arthroplasty (TKA) surgery is investigated using the dynamic time warping (DTW) algorithm. For this purpose, several volunteers who have problems with their knees are incorporated in a test before and after TKA surgery. Then, the data of gait analysis is collected and the data is compared with a reference using the DTW algorithm. The outcome results illustrate an improvement of 89%–97% by the proposed algorithm after TKA surgery. Therefore, patients can see improvement with high accuracy and very fast that result in more use this technique in TKR surgery.

## Graphical abstract

In this letter, the level of improvement in the human gait before and after total knee arthroplasty (TKA) surgery is investigated using the Dynamic Time Wrapping (DTW) algorithm. For this purpose, several volunteers who have problems with their knees incorporate in a test before and after TKA surgery, and data of gait analysis is collected. Then, the obtained data is compared with a reference using the DTW algorithm.

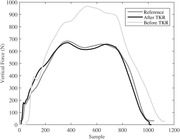

## 1 INTRODUCTION

Humans need two basic factors for gaiting first, the ability to stand and maintain balance, and second, the ability to perform alternating and sequential steps[1]. Although these two are basic needs, other factors are involved. Musculoskeletal systems always provide the opportunity for the joints to work properly, thereby allowing them to move properly. One of the most important joints in walking is the knee [2, 3]. The knee is the largest joint in the body that is made up of three bones: the lower part of the femur, the upper part of the tibia, and the patella. The lower end of the thigh has hinged movements on the leg, and the patella can move up and down in a groove‐like depression in front of the lower end of the thigh [4]. In the parts where these three bones are in contact with each other, their surface is covered with cartilage, and this slippery surface makes these three bones move easily on each other. There is also a thick, viscous fluid inside the knee joint called the synovial fluid, which slides over the cartilage and facilitates joint movement [5, 6]. When this joint is damaged due to knee injuries, it causes severe pain that may distract the person from even simple activities such as climbing stairs or walking [7]. The pain may even be present when sitting and sleeping. If medication cannot change the level of daily activities and also using a cane cannot help reduce the patient's problems, usually, the last step is to replace the knee joint with total knee arthroplasty (TKA). Although many patients have to replace the total of their knee by surgery, some of them replace one compartment of the knee which is called unicompartmental knee arthroplasty (UKA). Indeed, UKA is another surgical technique used for the treatment of osteoarthritis in one compartment of the knee, most commonly in the medial compartment [8]. Replacing the joint surfaces can solve the problem of knee pain and deformity and return the patient to daily activities [9].

Joint mobility and reducing pain are important goals of osteoarthritis treatment. There are some options for stepwise treatment that include exercise, weight loss, physiotherapy, analgesics, anti‐inflammatory drugs, intra‐articular steroids, hyaluronic acids, arthroscopic surgery, and in the final step, total joint replacement with follow‐up rehabilitation. All of the treatments are delivered by a range of healthcare professionals, including occupational therapists, physiotherapists, family physicians, and orthopedic surgeons. TKR is a final treatment for patients with severe pain and significant limitations. The first knee replacement was performed in 1968 [10]. After that, surgical techniques gradually improved and better artificial joints were made. Today, more than one million knee replacements are performed in the world every year. The important point before the surgery is that the patient should know what the benefits of this operation are for them and what problems cannot be solved and what problems can be made for them [11]. More than 90% of patients have significantly reduced pain after a knee replacement and are better able to perform daily activities. Of course, this surgery can never make your knee look exactly like a healthy knee[10]. After surgery, patients can do activities such as walking, swimming, cycling, driving, climbing stairs, or climbing. Activities such as brisk walking, hill climbing, skiing, tennis, and lifting objects weighing more than 25 kg are not very suitable for the artificial joint, and activities such as running, jumping, and impact sports are very dangerous for the artificial joint [12]. Even if the patients follow all the tips for good care of the artificial joint, after a while, the plastic part of the joint will wear away little by little. Certainly, intense physical activity or overweight speeds up this process [13].

In a study in [14], the defects and their compensation strategies after TKA in different conditions during the gait and the standing phase were investigated. Although TKA has the best standard for the treatment of advanced osteoarthritis, it still has side effects and consequences for the patient. Decline in physical function after exacerbation of osteoarthritis is severe but are not completely cured with TKA [14, 15]. Two other common consequences after TKA are a decrease in knee muscle strength and also ranges of motion (ROM), which are sometimes caused by the mechanics and design of the prosthesis [14]. Postpartum control after TKA also indicates the results. Although the inactive range of motion (before physiotherapy and home exercises to increase ROM) decreases after surgery, the same amount may be sufficient [14, 16]. Numerous factors such as decreasing muscle strength, declining self‐confidence in walking, bad habits, fear of movements can be the causes of walking problems. However, TKA can be effective [13]. Patients under the age of 65 should be considered as a suggestion to focus on the complications of TKA [17]. A large number of patients afraid of joint replacement however, if the accuracy of knee function before and after surgery can be calculated and illustrated, more patients tend to carry out this surgery. There are two essential techniques, mark‐based and mark‐independent, for measuring data on human gait. The marker‐based system is mounted on the body while the mark‐independent system does not need sensors or other markers on the body. Patients have to attach the sensors to their bodies in mark‐based and it is a very time‐consuming process. However, the mark‐independent system does not need to be attached to the body. Additionally, the mark‐based systems are free from a place despite the mark‐independent systems that are related to a place. Here, a mark‐independent system is chosen [18].

Here, evaluating the improvement of the knee joints in patients after TKA surgery is investigated using the DTW algorithm. For this purpose, several individuals with knee drawbacks are incorporated in a test, and data related to their motion analysis are collected before and after TKA surgery. The obtained data are compared with the human gait of a healthy person as a reference using the DTW algorithm. The results show that this algorithm shows a level of improvement with good accuracy and speed.

## 2 METHODOLOGY

### 2.1 The proposed method

**FIGURE 1:**
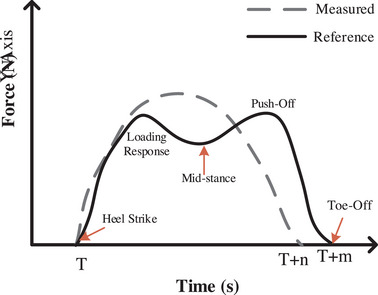
The output of force plate for a person with healthy and unhealthy knee, respectively, reference and measured.

A step of human gait for a healthy person is shown in Figure 1 (black solid plot) which consists of five main sections: heel strike, loading response, mid‐stance, push‐off, and toe‐off [19]. Patients who have problems with gait, miss at least one of the main sections. To evaluate the movement of patients after and before surgery, it is mandatory defining a function that performs a comparison between the obtained graph of the knee before and after surgery to a reference. As Figure 1 shows, the black solid plot is a reference graph for a volunteer with a healthy knee while the gray dash plot is a volunteer who has a defect of movement and suffers from problems in the knee. The function is an absolute value obtained from the difference between the reference and the measured value considering the performance of duration and variation of forces. The presented function is calculated by using dynamic time warping (DTW) [20, 21, 22, 23]. In time series analysis, DTW can reflect the similarity between two‐time sequences that are time‐varying [24].

Now, *F* is defined as follows:

(1) F(i,j)=infinitity

(2) $$\begin{array}{c} i=0,2,\text{…},n\\ j=0,2,\text{…},m\\ F(0,0)=0 \end{array}$$

In order to implement DTW, the two sequences *r* and *t* that are strings of discrete symbols are considered. Indeed, if the plot of reference and the measured data are defined with r[1:n], and t[1:m], respectively, then the distance between them can be defined as the following equation:

(3) $$d=r_{k}-t_{l}.$$

*F* that is defined DTW can be calculated by:

(4) $$\begin{array}{c} F(k,l)=d+min[F(k-1,l),F(k,l-1),F(k-1,l-1)] \end{array}$$

(5) $$\begin{array}{cc} k=1,2,\text{…} & ,n\\ l=1.2\text{…} & ,m. \end{array}$$

By implementation of the algorithm, DTW[i,j] is defined as the distance between r[1:n] and t[1:m] with the best alignment. The DTW algorithm produces a discrete matching between existing elements of one series to another while other methods allow continuous warping.

### 2.2 Ethics approval

The study was performed under the ethical standards as laid down in the 1964 Declaration of Helsinki and its later amendments or comparable ethical standards.

## 3 RESULTS

First, 18 volunteers are asked to participate in the test in which 56% were women and 44% were men. Their ages are between 58 and 78 with a mean of 68 ± 6.59 (Table 1). Also, their body mass index (BMI) was obtained, and these specifications are shown in Table 1. According to the data, 22% of the patients are obese and this obesity is an important factor for total replacement knee. Additionally, more women experienced the replacement of knee.

**TABLE 1:** Demographic and clinical features of the volunteers.

Number	Sex	age	BMI
1	Female	65	18.9
2	Female	60	21
3	Female	58	30
4	Female	66	20.3
5	Female	59	19
6	Male	70	22
7	Male	63	18.2
8	Female	72	23
9	Female	68	19.6
10	Male	74	21
11	Male	76	22.3
12	Male	65	18.9
13	Female	69	29
14	Male	77	30.5
15	Female	59	28
16	Male	78	23
17	Female	69	18
18	Female	76	21

**FIGURE 2:**
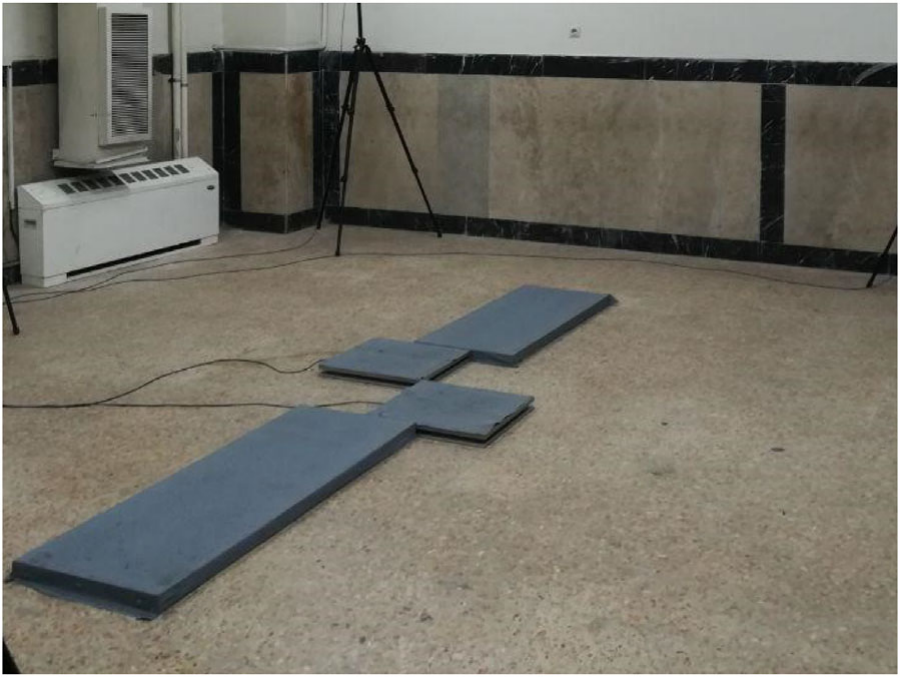
The measurement setup for collecting data on human gait.

These tests are performed in a room that is equipped with a force plate for gait analysis (Figure 2). This force plate can measure the forces developed during stepping, jumping, and other human actions. The maximum force is 3500 N in compression and 900 N when pull evenly distributed. Its resolution is 0.3 N and its size is 28 cm × 32 cm × 5 cm. The system is connected to a computer for saving data and also further processing. According to the subject under study, a correct method is selected to record the data with the help of a laboratory expert. Each test is recorded at least three times to collect accurate data. Every volunteer is asked to walk 10 steps with their foot, and all of the movements are saved for further processing. Indeed, these movements are compared to the reference movement by the proposed algorithm, and the mean of the error and improvement have been shown in Table 2 .

**TABLE 2:** Amount of improvements and also errors for several volunteers.

	Error	Error	Improvement
Volunteer	Before TKA	After TKA	(%)
1	1	0.06	94
2	1	0.09	91
3	1	0.11	89
4	1	0.07	93
5	1	0.05	95
6	1	0.04	96
7	1	0.08	92
8	1	0.03	97
9	1	0.11	89
10	1	0.09	91
11	1	0.03	97
12	1	0.08	92
13	1	0.05	95
14	1	0.06	94
15	1	0.04	96
16	1	0.08	92
17	1	0.06	94
18	1	0.03	97

The following points were considered in recording this data: First, the number of force plates (one or two) should be recorded on which the volunteers put each of their legs on the force plate. First, the number of force plates (one or two) should be recorded on which the volunteers put each of their legs on the force plate: for example, knowing that the volunteers put the first step with their right foot on the force plate #1. Second, each force plate records only the information of one foot and does not place the sample of both feet on one force plate, and finally, if the volunteers are not able to cross each force plate with one step, the force plates should adjust in other ways.

**FIGURE 3:**
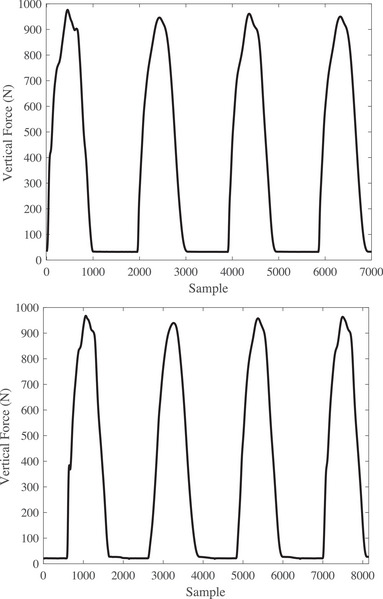
The raw data of the force plate for a patient before total knee arthroplasty (TKA) surgery. The top picture is for the right foot and the bottom is for the left foot.

**FIGURE 4:**
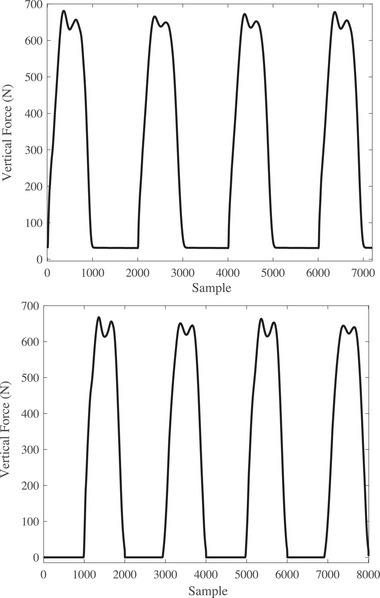
The raw data of the force plate for a patient after total knee arthroplasty (TKA) surgery. The top picture is for the right foot, and the bottom is for the left foot.

Figure 3 illustrates four gait signals per subject of the force plate for the left and right feet for a volunteer before TKA surgery. As it can be seen, the volunteer first puts the right foot and then the left foot. Moreover, the volunteer did not put both the feet simultaneously. Also, there is high‐frequency noise whose value can be removed by cutting the proper movement. Additionally, the volunteer put his left foot twice that first time is not a proper movement and the obtained data also was removed. Figure 4 shows the raw data for the patient after TKA surgery. Although the movement has improved, the figure needs to be preprocessed before using the DTW algorithm.

After the analysis and completion of the work in the laboratory, a data file is obtained. This file is prepared as follows:

1. Cutting the beginning and end of the file until a few steps of movement remain in the parts that have been recorded with high quality.
2. Removing gaps

**FIGURE 5:**
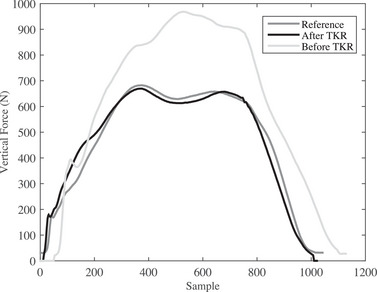
The obtained results of the force plate for a patient before and after arthroplasty (TKA) surgery for the right foot. The black line is a reference plot that is related to the gait of a person with a healthy knee.

The obtained data before and after TKA surgery as well as the reference plot are shown in Figure 5 for the right foot. As earlier mentioned, the reference plot is extracted from a healthy knee in human gait. The obtained results illustrate many differences in the measured force between the reference plot and before TKA. However, the differences are low after TKA. Because the maximum difference was related to before TKA, all data were normalized to before TKA. After TKA surgery, the volunteer performed a movement similar to the reference. By calculating the distance by DTW algorithm and normalizing, the value of difference gains 0.06 after TKA surgery while this value is 1 before TKA. Also, Figure 5 illustrates that the volunteer carried out heel strike and toe‐off correctly for both tests: before and after TKA. Nonetheless, three other major areas consisting of loading response, mid‐stance, and push off have not been done correctly before TKA. After surgery, the volunteer correctly fulfilled a step that consist of heel strikes, toe‐off, loading response, mid‐stance, and push‐off. The test was performed by several volunteers and similar results were obtained. Table 2 illustrates 18 volunteers before and after TKA, and all of them have an improvement between 89% and 97% in their human gait by the DTW algorithm. Also, the mean improvement is 95.6%, and as a result, significant improvements were seen in terms of reduction of pain and improved function for patients who underwent TKR surgery.

As earlier mentioned, the algorithm measures the difference between two movements together, even for movements with several different samples. As Figure 5 shows, three movements of reference, afterTKR, and BeforeTKR have 1025, 1010, and 1180 samples, respectively. It is mandatory to calculate the difference by complex methods without the DTW algorithm. As a result, it takes a lot of time. However, the difference can be calculated by the proposed algorithm with less time and it is not time‐consuming. Therefore, DTW can be used to investigate the improvements after TKA surgery for people with knee problems.

## 4 DISCUSSION

Generally, patients who undergo TKR surgery for osteoarthritis have substantial improvements in terms of reduction of pain and also improved function. A comparison of previous studies that reported preoperative and postoperative outcome scores for patients of TKR showed that the procedure was effective [25]. The mentioned studies included patients of various ages and used a variety of prostheses and techniques to implant the device [26, 27]. TKR was effective in all of the studies [26, 27]. Furthermore, unicompartmental knee replacement (UKR) surgery seems to be as effective as TKR surgery for people who have the indications for it. This is a subset of people who have osteoarthritis of the knee because for UKR to be indicated, only one compartment of the knee can be affected. Patients who undergo UKR surgery seem to stay in the hospital shorter and there are faster recovery times than patients who have TKR surgery [8]. There are some important factors such as obesity, age, gender, prosthesis design, and surgical techniques that predict TKR outcomes. However, none of these factors have been shown to predict pain or function consistently across studies [28]. The obtained results in the result section illustrate that patients have usually experienced obesity and older age for more cases. For example, all cases are between 58 and 78 years old. Four volunteers are obesity that 75% of them is female. The results illustrated that the obesity and sexuality are more effective than other factors for TKR surgery.

Obtaining human movements is one of the most significant research methods in biomechanics. In the past, much attention was given to discovering diseases related to human skeletons and muscles based on movements. Recently, human movement and also rehabilitation [29, 30, 31, 32, 33] have been used to prevent skeletal and muscular diseases. Although motion analysis is extremely useful in hospitals and medical centres, the use of gait analysis in these places is tremendously limited and has not grown significantly [34]. Therefore, the use of motion analysis methods in these centres is especially valuable because it is exceedingly useful for solving the problem related to a human gait, and on the other hand it will save money and time in collecting, processing, and interpreting data. By using the proposed algorithm here, patients will see the results of their improvement as soon as possible and try to improve it.

In general, there are many ways to measure information about human gait. These methods can be divided into mark‐based and marks‐independent. One of the most basic technical factors in the study of human gait is how to move the skeleton of the body. It is possible to use markers mounted on the body [18]. The methods are called marks‐based methods and are installed on the body to receive information. Currently, most of the proposed methods in the field of human gait have been marks‐based that require a series of sensors that can be installed on different parts of the human body to obtain accurate three‐dimensional information about the human gait [35]. The major problem with mark‐based systems is that it takes time to prepare the candidates for the test. However, the mark‐based methods own very accurate indications and are closer to the actual values [18]. When the marks are installed on the body and the person begins gait, these signs also begin to move, and the movement of the lower bones can be understood from the movement of these signs [36]. The problem here is that the movement of the skin of the body is accompanied by the movement of the following bones, which leads to the wrong movement of the marks, and as a result, the accuracy of the measurement is reduced [36]. Other methods are marks‐independent, in that no sign or sensor is installed on the body. Therefore, the patient never needs to be prepared for the installation of these systems on their body, and as a result, this method saves time for patients. On the other hand, these methods are less accurate than marks‐based methods [37]. In the first method, there is a large amount of data for processing from which the desired properties must be extracted, whereas in the marks‐based methods, the desired information was provided concerning that particular location [36]. Here, data is collected from a marks‐independent method that results in performing the test in the fastest possible way by the candidates.

It should be noted that using the algorithm for the force plate is good and doctors can give the result with a quantity value to patients and encourage them for more attempts. However, the patients have to refer to the clinic for taking the tests, and the process is very time‐consuming. The proposed algorithm in a wearable system can be used in the future. Indeed, a combination of the DTW and wearable sensors can be employed at home for patients. Wearable sensors can be mounted on the joints to measure the angular variations of joints, and the proposed algorithm can calculate the difference between the reference and the measured movement. As a result, the patient saves time and cost, and they will obtain further guidance when they call the doctors.

## 5 CONCLUSIONS

Here, a proposed method for calculating the level of improvement in patients after TKA surgery was performed based on the DTW algorithm. To do this, first, several volunteers with serious problems with their knees were asked to participate in the test. Their data of gate analysis were recorded before and after TKA surgery. Then, the data were compared with the data of a healthy person as a reference by the proposed algorithm of DTW. The obtained results showed an improvement of 89%–97% for patients who participated in TKA surgery with high accuracy and speed. Therefore, the algorithm can give more promise to people who endure knee problems for TKR surgery.

## CONFLICT OF INTEREST STATEMENT

The authors declare no conflicts of interest.

## Data Availability

The data that support the findings of this study are available from the corresponding author upon reasonable request.
